# Machinability of Luxury Vinyl Tiles during Plain Milling Using a Helical Cutter

**DOI:** 10.3390/ma12132174

**Published:** 2019-07-06

**Authors:** Zhaolong Zhu, Pingxiang Cao, Xiaolei Guo, Xiaodong (Alice) Wang, Fan Zhang, Yu Gao

**Affiliations:** 1 College of Materials Science and Engineering, Nanjing Forestry University, Nanjing 210037, China; 2 Department of Wood and Forest Sciences, Laval University, Quebec, QC G1V 0A6, Canada

**Keywords:** LVT, PCD cutting tool, helix cutting, orthogonal design, optimization

## Abstract

In order to better provide a theoretical basis for the machining of luxury vinyl tiles, a helical milling experiment was conducted by using diamond cutting tools, and special attention was given to the trends of cutting force and surface roughness in respect to tool geometry and cutting parameters. The results showed that the resultant force was negatively correlated to the helix angle and cutting speed, but positively correlated with the cutting depth. Then, that the surface roughness increased with a decrease of the helix angle and an increase of cutting depth, while as cutting speed raised, the surface roughness first declined and then increased. Thirdly, the cutting depth was shown to have the greatest influence on both cutting force and surface roughness, followed by helix angle and cutting speed. Fourth, the contribution of cutting depth only was significant to cutting force, while both the helix angle and cutting speed had insignificant influence on the cutting force and surface roughness. Finally, the optimal cutting conditions were proposed for industrial production, in which the helix angle, cutting speed and cutting depth were 70°, 2200 m/min and 0.5 mm, respectively.

## 1. Introduction

Luxury vinyl tile (LVT), as a new engineering material, has been extensively used in flooring and wall decoration, because of its environmental protection, ultra-wear-resistance, and its being waterproof [1]. LVT is mainly made from the mixture of calcium carbonate, polyvinyl chloride and other auxiliary materials, then formed into panels by applying pressures, 4–17 MPa, and temperatures, 150–170 °C, in a hot press. Finally, the wear layer and colorful printing layer are directly laminated to the panels by heating in the calendaring process [2,3].

Compared with the traditional engineering materials used in flooring, LVT is a hard-to-cut material. The calcium carbonate contained in LVT has high hardness, causing tool wear, and leading to shorter service life of cutting tools. Diamond cutting tools have been extensively used to improve production efficiency [4], due to their superior properties, such as high hardness [5], low friction coefficient [6], high elastic modulus [7] and low thermal expansion coefficient [8].

In the literature, the main research subjects during different materials processing have always been the cutting force and roughness of the machined surface, which directly affect the energy consumption of the machine [9], tool design [10], and smoothness of the machined surface [11]. Meanwhile, the cutting force and the surface roughness are sensitive to the cutting parameters, tool geometries and so on, as shown by the following references. Pramanik et al. [12] studied the changes in cutting force and surface roughness when turning electroless-nickel plated die with a diamond cutting tool, wherein they determined that the cutting forces show an increasing trend with the increased spindle speed, feeding rate and cutting depth, while the contribution of cutting depth to surface roughness is insignificant, and the surface roughness first decreases and then increases with the increasing spindle speed. In related studies, Cao et al. [13] and Zhu et al. [14,15], investigated the machinability of the stone-plastic materials, a composite similar to LVT, and their results showed that the cutting forces and surface roughness show a similar trend under different cutting forces, where they all are positively correlated with cutting depth, but negatively related to cutting speed and rake angles of cutters. Taking cutting force and surface roughness as evaluating indicators, the cutting parameters were optimized by Yalcin et al. [16], wherein milling steel, and the optimum selection of cutting parameters importantly contributed to the improvement of the economic benefit and productivity [17].

LVT is a difficult to machine material due to its special material composition, according to previous research [18]. The calcium carbonate contained in the LVT has high hardness, which leads to a lower cutting stability and severe tool wear, and this directly affects the cutting quality of the machined surface. Helical milling is one of the most popular cutting methods, where the cutting edge attached to the rotating axis traverses a helical trajectory and forms a cylindrical surface [19]. Compared with the common straight-tooth cylindrical milling, helical milling is recommended for improvement of machining efficiency, cutting quality and stability, and to reduce cutting abrasion [20,21,22]. Although helical milling has a wide application field for processing various materials [23], it is rarely used in LVT machining. Meanwhile, for LVT as a new engineering material, there is still no report that focuses on its machinability during helical milling. How to scientifically select the optimal cutting parameters and tool geometries has been an urgent problem for LVT enterprises.

In this study, a series of helical milling experiments were carried out, mainly describing the changes in cutting force and roughness of machined surface in terms of tool geometries and cutting parameters. Based on the results of this analysis, the optimal cutting conditions in terms of cutting quality, energy consumption and productivity were determined, which aims to provide guidance for helical milling adopted in LVT machining.

## 2. Materials and Methods

**Figure 1:**
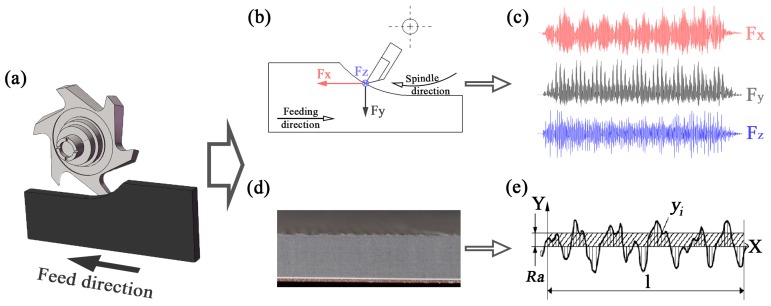
Milling experiment: (**a**) Helical milling; (**b**) measurement of cutting forces; (**c**) waveform of cutting forces; (**d**) machined surface of Luxury vinyl tile (LVT); (**e**) surface roughness.

As shown in Figure 1a, up milling was conducted in a commercial computerized numerical control (CNC) machining center (MGK01, Nanxing Machinery Co., Ltd., Guangzhou, China) under dry conditions with a constant feed rate of 15 m/min, in which the LVT (Table 1) was milled by helical diamond cutting tools (Table 2). As shown in Figure 1a–c, the cutting forces generated in helical milling, namely *F_x_*, *F_y_* and *F_z_*, were acquired by a three component piezo-electric dynamometer (9257B, Kistler Group, Winterthur, Switzerland) allowing measurements from −5.0 to 5.0 kN, and a charge amplifier (Kistler 5070A) with a rate of 7100 samples per second. Finally, the dynamic cutting forces were processed and analyzed by DynoWare software (Version 2.6.5.16, Kistler Group). To better understand the changes in cutting force, the resultant force was considered and calculated as shown in Equation (1):

(1) $$F_{R}=\sqrt{F_{x}{}^{2}+F_{y}{}^{2}+F_{z}{}^{2}}$$

where *F_R_* was defined as the resultant force, *F_x_*, *F_y_* and *F_z_* were the component forces parallel to the feeding direction, perpendicular to the feeding direction, and parallel to the direction of the cutter axis, respectively.

As displayed in Figure 1d,e, surface roughness, *Ra* was taken as the evaluation index for the smoothness of the machined surface, which was measured by using a surface profiler (S-NEX001SD-12, Tokyo Seimitsu Co., Ltd., Tokyo, Japan), and calculated by the software ACCTee. Meanwhile, a scanning electron microscope (SEM) (Quanta 200, FEI Co., Ltd., Hillsboro, OR, USA) was used to observe the machined surface of LVT.

As given in Table 3, three factors, including helix angle, cutting speed and cutting depth were identified, and the range of each factor for the study was determined from the LVT industrial processing. In this work, Taguchi methods were used, which is a kind of design method with multiple factors and multiple levels, based on the orthogonality selected from a comprehensive test of some representative points test [24,25]. The cutting force and surface roughness were measured 3 times in each combination of cutting parameters according to the orthogonal Latin square design table L_9_ (3^4^) [26], and the average values were obtained.

## 3. Results and Discussion

### 3.1. Range Analysis of Milling Force

Table 4 shows the range analysis results of resultant force; R represents the difference between the maximum and minimum values of K_i_ of each factor. The higher the R value is, the greater influence of the factor on the results [24,25,26]. According to the comparison of values of R_I_, R_II_ and R_III_ (R_III_ = 60.832 > R_I_ = 13.636 > R_II_ = 1.509), factor III (milling depth) had the greatest influence on cutting force, while factor II, the spindle speed, had the least impact on cutting force. Thus, during helical milling of LVT, the cutting depth had the greatest effect on the resultant force, followed by the helix angle and the spindle speed.

Lower cutting force means greater cutting stability. By taking the resultant force as the evaluation index: The minimum cutting force generated can then be selected by choosing the optimal combination of cutting parameters. Based on value of K_i_, it can be concluded that I_3_, II_3_, III_1_ were the optimal combination of cutting parameters; e.g., the helix angle was 70°, the cutting speed was 2640 m/min and the cutting depth was 0.5 mm.

**Figure 2:**
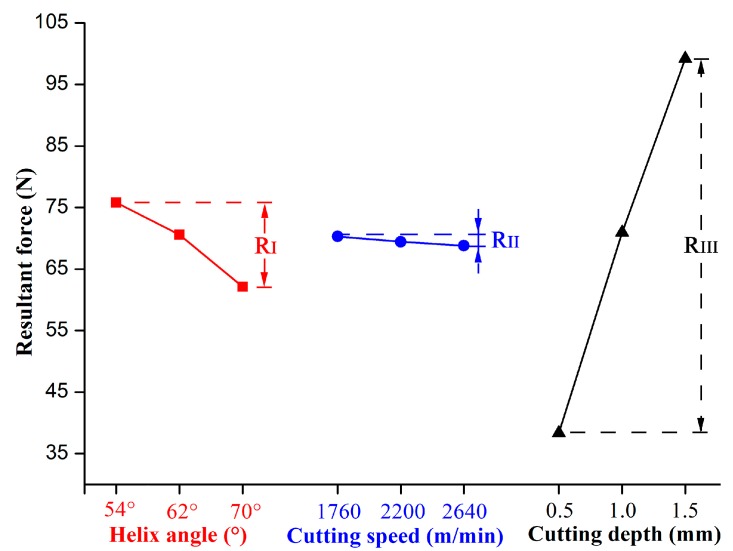
Influence of cutting conditions on resultant force.

**Figure 3:**
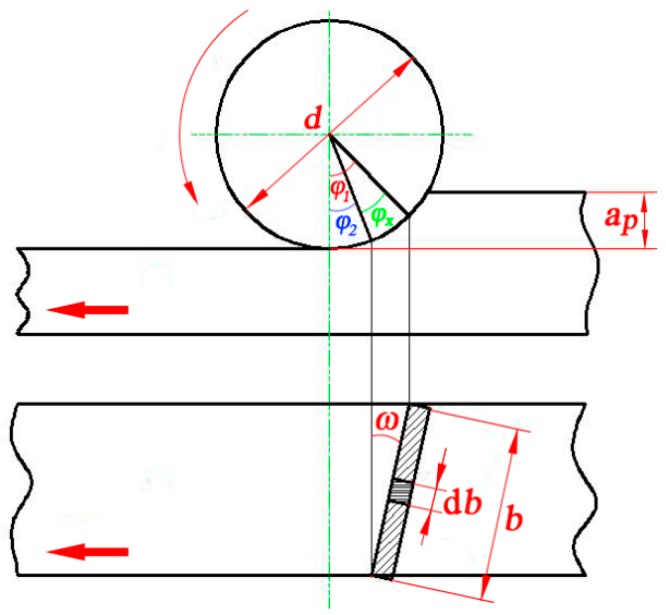
Change of chip width during helical milling process.

Figure 2 illustrates the trend of resultant force under different cutting conditions; it can be found that the cutting force decreased with the increase of the helix angle. As shown in Figure 3, the chip width was continuously changed during the helical milling. When the cutting tool edge bites into the workpiece, the chip width increased from zero to maximum, then the chip width gradually decreased to zero. Finally the chip was removed by the rake face of the cutter. For more details, the expressions for the relationship between chip width and the rotating and helix angles of the cutter are given in Equations (2) and (3). With an increase of the helix angle, the chip was more easily removed from the cutting layer, so that the cutting force decreased with the increase of the helix angle.

(2) $$db=\frac{\frac{d}{2}d\varphi }{\sin\omega }=\frac{d}{2\sin\omega }d\varphi$$

(3) $$b=\int_{\varphi_{2}}^{\varphi_{1}}\frac{d}{2\sin\omega }d\varphi =\frac{d(\varphi_{1}-\varphi_{2})}{2\sin\omega }=\frac{d\cdot \varphi_{x}}{2\sin\omega }$$

where $\varphi_{1}$ and $\varphi_{2}$ are the rotating angles of the cutter, *ω* is the helix angle of the cutter, *b* is the chip width, and *d* is the tool diameter with a constant of 140 mm.

**Figure 4:**
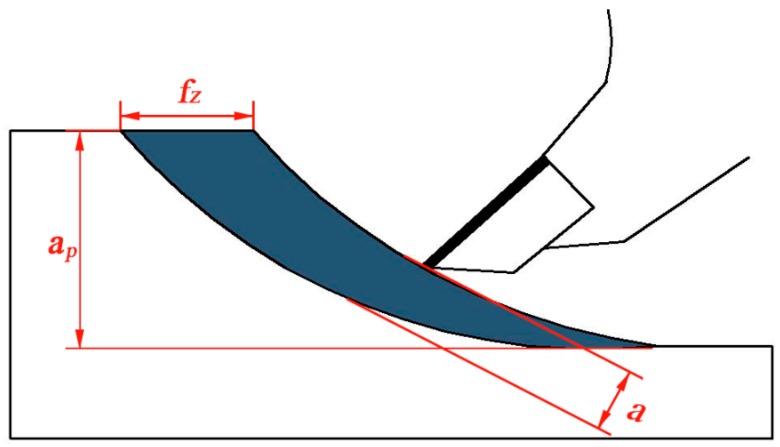
Schematic diagram of feed per tooth, cutting depth and chip thickness in the up milling.

It can also be seen from Figure 2 that the cutting force decreased slightly with the increase of cutting speed, but increased with an increase in cutting depth. The trends of cutting forces with different cutting conditions are similar to results obtained by Zhu et al., when stone-plastic material (a composite material with structure like LVT) was machined by PCD cutters during orthogonal cutting [14,15]. Figure 4 is the schematic diagram of feed per tooth and chip thickness during helical up milling. When the cutting edge bit into the workpiece, the chip thickness was zero. The chip thickness would continually increase to a maximum until the chip was separated from workpiece. The equations of feed per tooth and chip thickness with different parameters are expressed as Equations (4) and (5). It can be found that a decreasing cutting speed or increasing cutting depth enhances the feed per tooth and average chip thickness, which results in more removal of chips; i.e., the increased load acting on the cutter resulted in an increase in cutting force. Therefore, the cutting force showed an increasing trend with the decreased cutting speed, but increased cutting depth.

(4) $$f_{z}=\frac{\pi f_{n}d}{60v_{c}z}$$

(5) $$a_{av}=f_{z}\cdot \sqrt{\frac{a_{p}}{d}}$$

where *f_z_* is defined as feed per tooth, *f_n_* stands for feeding rate, *v_c_* means cutting speed, *a_p_* is cutting depth, *z* is the tooth number, *a_av_* stands for the average chip thickness, and *d* means the diameter of the cutting tool.

### 3.2. Analysis of Variance (ANOVA) of Cutting Force

The ANOVA was used to prove the prominence of each factor, which is judged by a significance level of α = 0.05 (95% confidence level, F_0.05_ = 19.00). If the F value of a factor is less than the F_0.05_ = 19.00, it can determine that the factor has an insignificant effect on the result, which is otherwise significant [24,25,26]. As listed in Table 5, F_I_ = 1.832 < F_0.1_ = 9.00, and F_II_ = 0.022 < F_0.1_ = 9.00 show that the contributions of helix angle and cutting speed on the cutting force were insignificant, while F_III_ was equal to 35.817, which exceed the value of F_0.05_, namely 19.00. Thus, only cutting depth has significant influence on the cutting force.

### 3.3. Range Analysis of Surface Roughness

**Figure 5:**
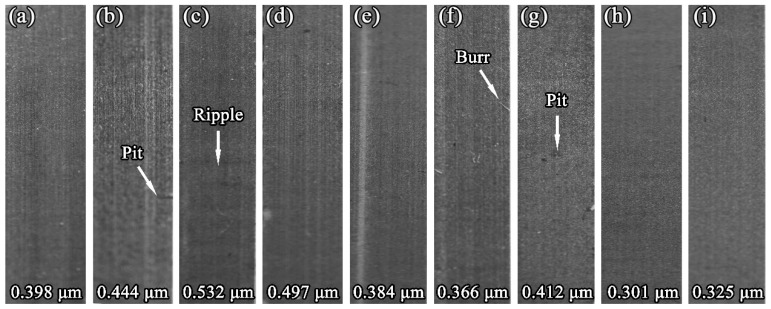
Damage morphology of the machined surfaces (the cutting conditions of **a**–**i** are consistent with the No. 1–9 in Table 6).

**Figure 6:**
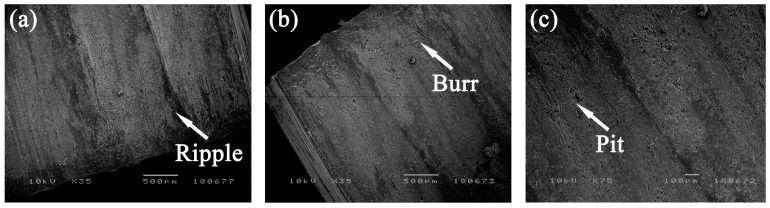
SEM micrograph of ripple (**a**), burr (**b**) and pit (**c**) generated under the cutting conditions of 3, 6 and 7, respectively.

The damage morphology of the LVT surface machined in different cutting conditions was shown in Figure 5. Ripples were found on the machined surface when the helix angle was 54°, cutting speed was 2640 m/min and the cutting depth was 1.5 mm. There were also some visible waved surfaces observed on the workpiece (Figure 6a). When the helix angle was 70°, cutting speed was 1760 m/min and the cutting depth was 1.5 mm (Figure 6b), burr could be seen on the machined surface, and was loosely connected to the machined surface. Pits were observed on the machined surface for a 54° helix angle, 2200 m/min cutting speed and 1.0 mm cutting depth, and for a 70° helix angle, 1760 m/min cutting speed, and 1.5 mm cutting depth. As in Figure 6c, it was found that the machined surface with pits looked unsmooth. Meanwhile, there was no obvious damage found on the machined surface during other cutting conditions. In order to better understand how the cutting conditions affected the cutting quality, the range analysis results of the machined surface roughness were tabulated in Table 6. Based on comparing the R-values of various factors, it was found that R_I_ = 0.112 > R_III_ = 0.088 > R_II_ = 0.060. Therefore, cutting depth had the greatest influence on surface roughness, followed by helix angle and cutting speed. Considering the surface roughness as the evaluation index, the lower the surface roughness is, the better the cutting quality. Thus, the optimal cutting condtion with the lowest surface roughness were chosen, which were I_3_, II_2_, III_1_, namely a helix angle of 35°, a cutting speed of 2200 m/min and a cutting depth of 0.5 mm.

**Figure 7:**
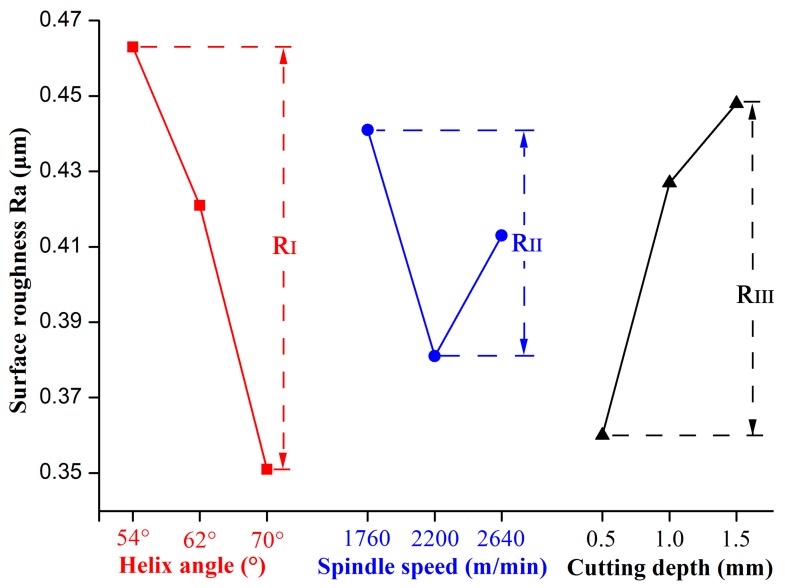
Influence of cutting conditions on surface roughness.

Figure 7 shows the influence of the cutting parameters on the surface roughness and it indicates that the surface roughness decreased with the increasing of the helix angle. This was because as the helix angle increased, the cutting force decreased, and the cutting process became more stable. Hence, the cutting quality was improved with the increased helix angle. Meanwhile, it was found that the surface roughness first decreased with the increase of cutting speed, which agrees with the most recent research results [12,15]. However, as cutting speed continued to grow, the surface roughness showed an increasing trend. This abnormal change was mainly due to the cutting heat generated during machining. LVT is mainly made from calcium carbonate and PVC, and the PVC was easy to degrade in the high temperature environment. The more the cutting speed increased, the higher the cutting temperature that was produced. Thus, the poor quality of the machined surface can be associated with temperature increase in the cutting zone [15,18]. Finally, it can be concluded from Figure 5 that the surface roughness increased with the increase of cutting depth, and the changes of LVT’s surface roughness had the same trend to that of stone-plastic material [14]. As described in Section 3.1, the increase of cutting depth enhanced the removal volume per unit time of the cutting tool, which reduced the cutting stability, so the cutting quality decreased with the increase of cutting depth.

### 3.4. ANOVA of Surface Roughness

According to the variance test results (Table 7), F_I_ = 2.375, F_II_ = 0.625, and F_III_ = 1.625, were all less than F_0.1_ = 9.00. Thus, whether it was helix angle, cutting speed or cutting depth, their effects on the surface roughness of the LVT were not significant.

### 3.5. Optimization of Tool Geometries and Cutting Parameters

As mentioned above, taking the cutting force and surface roughness as optimizing targets, the optimum parameters are I_3_, II_3_, III_1_ (70° helix angle, 2640 m/min cutting speed, 0.5 mm cutting depth) and I_3_, II_2_, III_1_ (70° helix angle, 2200 m/min cutting speed, 0.5 mm cutting depth), respectively. However, the cutting speed had no significant effect on either cutting force or surface roughness. In that case, considering the productivity, energy consumption and product quality, the cutting speed should be lower but still appropriate for a satisfying result, because lower cutting speed reduces severity of tool wear and powder consumption of the machine. Therefore, the optimal cutting conditions are proposed as follows: 70° helix angle, 2640 m/min cutting speed, and 0.5 mm cutting depth. For those optimal conditions, the resultant force and surface roughness are 36.221 N and 0.301 μm, respectively, coinciding with the results of the orthogonal experiment (Table 4 and Table 6), where the best cutting quality was obtained. According to previous research [18], the main wear patterns of diamond cutting tools when milling LVT are cracking, chipping and flanking, and the wear mechanisms of diamond cutter are abrasive and adhesive wear. All in all, in order to improve the surface quality of LVT, productivity and energy consumption during processing, the optimal combination of cutting conditions is a 70° helix angle, 2640 m/min cutting speed and 0.5 mm cutting depth.

## 4. Conclusions

Based on the orthogonal experiment design, the cutting force and surface roughness were focused in terms of cutting parameters and tool geometry, when LVT was milled by helical diamond cutting tools. The conclusions can be drawn as follows:

(1) Resultant forces showed a decreasing trend with the increase of helix angle and cutting speed, but it increased with an increase in cutting depth.
(2) Roughness of the machined surface increased with a decreased helix angle and increased with more cutting depth. However, the surface roughness first declined and then increased as the cutting speed continuously increased.
(3) Cutting depth has the greatest influence on both cutting force and surface roughness, followed by helix angle and cutting speed. However, only cutting depth had a significant effect on the cutting force. The contribution of both helix angle and cutting speed were insignificant to cutting force and surface roughness.
(4) The optimal cutting conditions when milling LVT were obtained and verified to be as follows: 70° helix angle, 2200 m/min cutting speed and 0.5 mm cutting depth.

## Figures and Tables

**Table 1:** Material properties of LVT.

Density	Modulus of Elasticity	Thermal Conductivity	Friction Coefficient
1380 kg·m^−3^	2752 MPa	0.17 W·m^−1^·K^−1^	0.45

**Table 2:** Geometries and properties of polycrystalline diamond (PCD) inserts.

PCD Inserts	Tool Geometries			Material Properties		
	Helix Angle	Rake Angle	Clearance Angle	Modulus of Elasticity	Thermal Conductivity	Hardness
1	54°	10°	8°	800 GPa	560 W·m^−^^1^·K^−^^1^	8000 HV
2	62°	10°	8°			
3	70°	10°	8°			

**Table 3:** Cutting parameter including helix angle (°), cutting speed (m/min) and depth (mm), and their levels.

Levels	Factors		
	Helix Angle (I)	Cutting Speed (II)	Cutting Depth (III)
1	54	1760	0.5
2	62	2200	1.0
3	70	2640	1.5

**Table 4:** Range analysis result of resultant force (N) under different helix angle (°), cutting speed (m/min) and depth (mm).

Number	Helix Angle (I)	Cutting Speed (II)	Cutting Depth (III)	*F_R_*
1	54	1760	0.5	44.996
2	54	2200	1.0	72.307
3	54	2640	1.5	110.055
4	62	1760	1.0	78.153
5	62	2200	1.5	99.778
6	62	2640	0.5	33.906
7	70	1760	1.5	87.784
8	70	2200	0.5	36.221
9	70	2640	1.0	62.444
K_1_	75.786	70.311	38.374	-
K_2_	70.612	69.435	70.968	-
K_3_	62.150	68.802	99.206	-
R	13.636	1.509	60.832	-

Note: K_i_ represented the average value of level i (1–3) of a factor, and R meant the difference between the maximum and minimum values of K_i_ of each factor.

**Table 5:** ANOVA of cutting force with R-squared of 0.94.

Factors	SS	DOF	F	Prominence
Helix angle (I)	284.333	2	1.832	Insignificant
Cutting speed (II)	3.446	2	0.022	Insignificant
Cutting depth (III)	5560.164	2	35.817	Significant
Error	155.24	2	-	-
Total	6003.183	8	-	-

**Table 6:** Range analysis result of surface roughness (μm) under different helix angles (°), cutting speed (m/min) and depth (mm).

Number	Helix angle (I)	Cutting speed (II)	Cutting depth (III)	*Ra*
1	54	1760	0.5	0.398
2	54	2200	1.0	0.444
3	54	2640	1.5	0.532
4	62	1760	1.0	0.497
5	62	2200	1.5	0.384
6	62	2640	0.5	0.366
7	70	1760	1.5	0.412
8	70	2200	0.5	0.301
9	70	2640	1.0	0.325
K1	0.458	0.436	0.355	-
K2	0.416	0.376	0.422	-
K3	0.346	0.408	0.443	-
R	0.112	0.060	0.088	-

**Table 7:** ANOVA of surface roughness with R-squared of 0.95.

Factors	SS	DOF	F	Prominence
Taper angle (I)	0.019	2	2.375	Insignificant
Cutting speed (II)	0.005	2	0.625	Insignificant
Cutting depth (III)	0.013	2	1.625	Insignificant
Error	0.010	2	-	-
Total	0.047	8	-	-

## Nomenclature

*F_R_*:	Resultant force (N)
*F_x_*:	The component force parallel to the feeding direction (N)
*F_y_*:	The component force perpendicular to the feeding direction (N)
*F_z_*:	The component force parallel to the direction of the cutter axis (N)
*Ra*:	Surface roughness (μm)
*f_z_*:	Feed per tooth (mm)
*f_n_*:	Feeding rate (m/min)
*v_c_*:	Cutting speed (m/min)
*ap*:	Cutting depth (mm)
*z*:	Tooth number of cutter
*a_av_*:	Average chip thickness (mm)
*d*:	Diameter of cutter (mm)
*ω*:	Helix angle of cutter (°)
*b*:	Cutting width (mm)
